# Supplementary material for: TFEB regulates murine liver cell fate during development and regeneration
Source: Nat Commun. 2020 May 18;11:2461. doi: 10.1038/s41467-020-16300-x (PMC7235048; doi:10.1038/s41467-020-16300-x)
Supplement: Supplementary file 1 — Supplementary Information [file 41467_2020_16300_MOESM1_ESM.pdf]

Supplementary information

TFEB regulates liver cell fate during development and regeneration

Pastore et al.

Supplementary Fig. 1

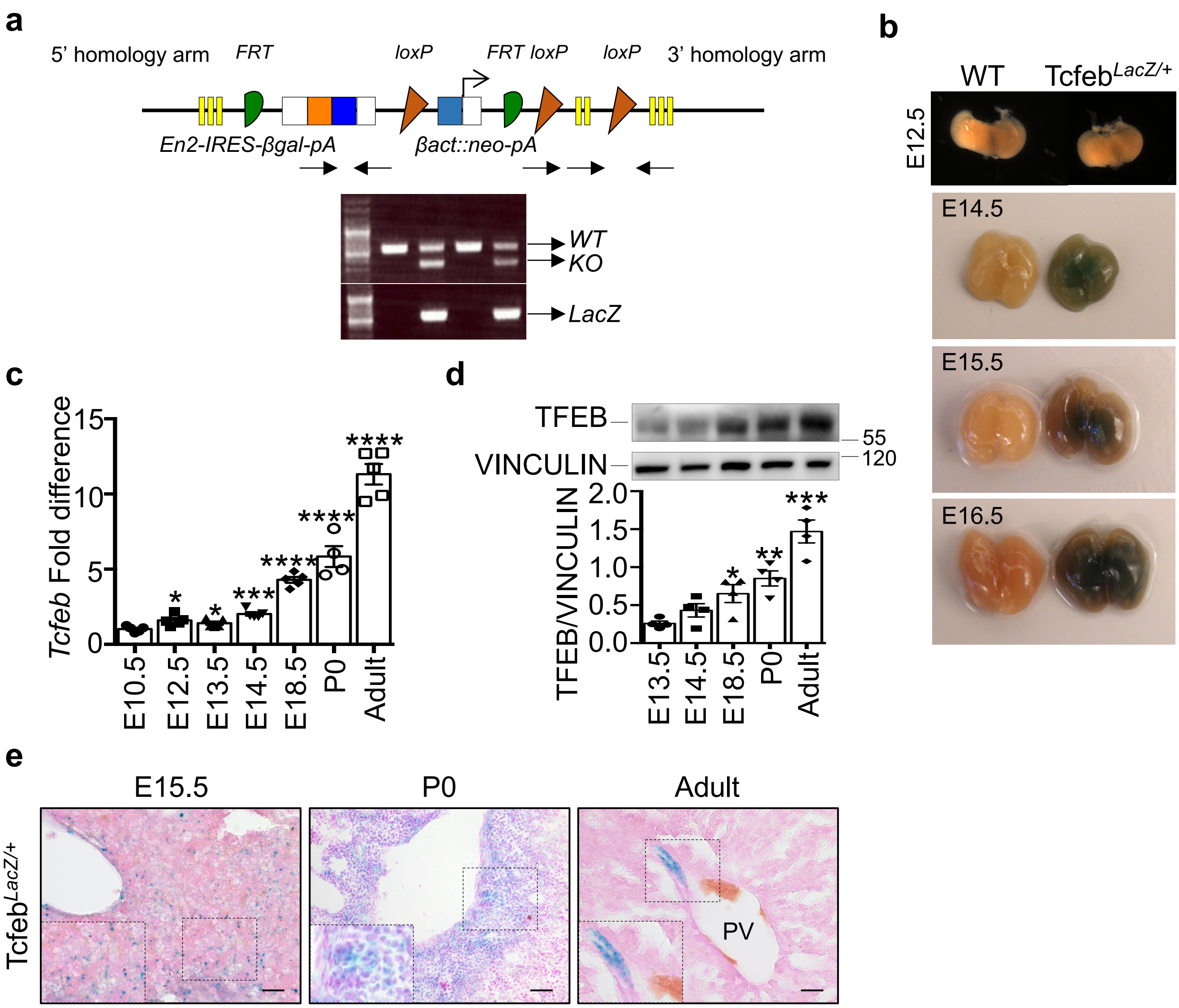

Supplementary Fig. 1. TFEB expression increases during liver development. **a** Schematic representation of the *Tcfeb*^{LacZ} mouse line. **b** Representative images of whole-mount liver from WT and *Tcfeb*$^{LacZ/+}$ embryos at the indicated stages after X-Gal staining. **c,d** Expression analysis of *Tcfeb* during liver differentiation. Bars show *Tcfeb* mRNA (*n*=5) (**c**) and protein (*n*=4) (**d**) levels in livers isolated from mice at the indicated stages. Data are represented as mean ± SEM. *$p \leq 0.05$; ***$p \leq 0.001$; ****$p \leq 0.0001$ two-tailed Student's t-test compared to E10.5. **e** X-Gal staining of liver sections from *Tcfeb*$^{LacZ/+}$ mice at the indicated stages. PV= portal vein. Scale bar 50 μm. Source data are provided as a Source Data file.

Supplementary Fig. 2

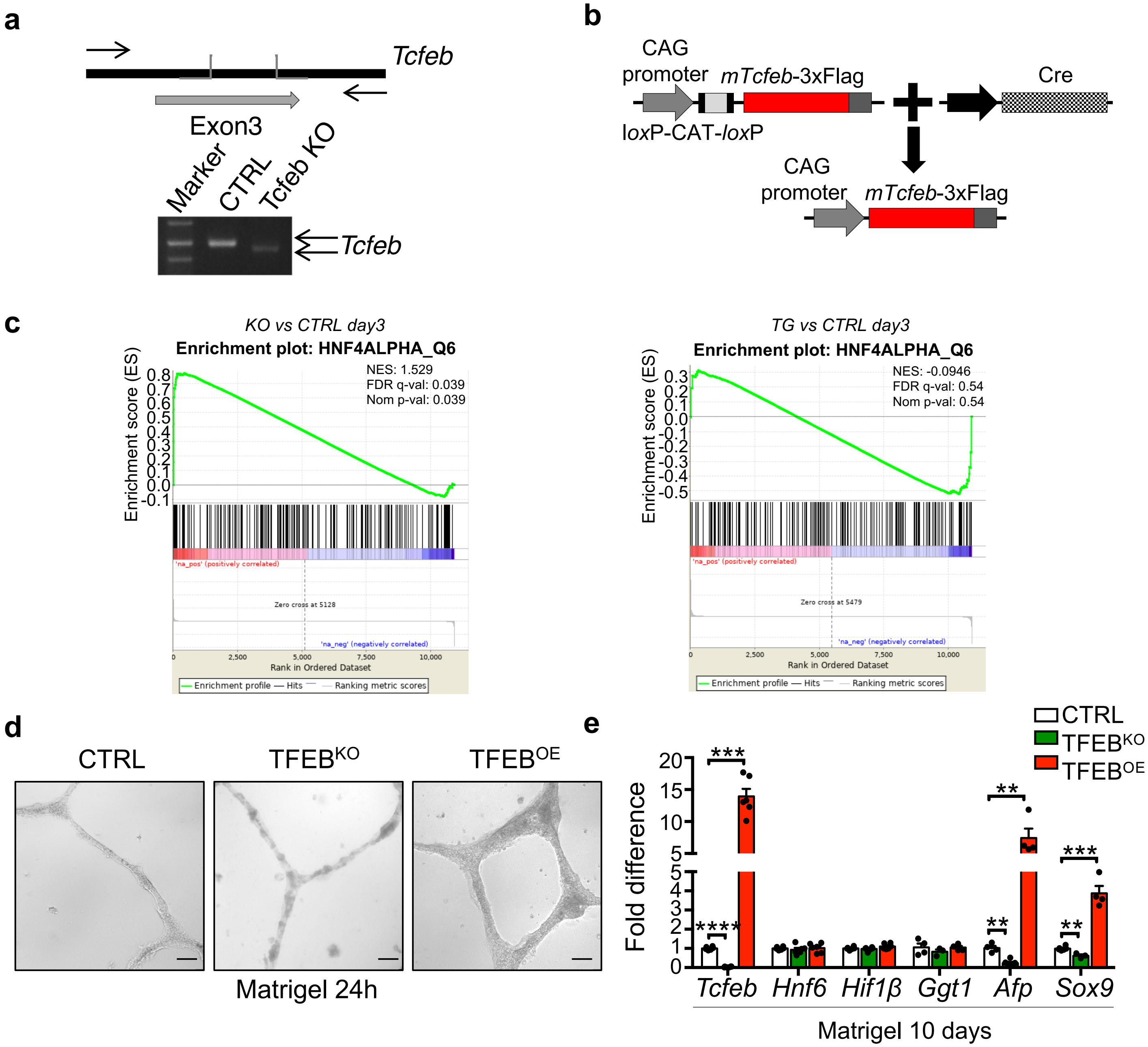

Supplementary Fig. 2. TFEB overexpression alters hepatoblast (HB) differentiation in hepatocytes. **a,b** Representation of the strategy used for the generation of TFEB^{KO} (**a**) and TFEB^{OE} (**b**) HBs. **c** Enrichment plot (GSEA) analysis showing differential expression of HNFα-targets in TFEB^{KO} and TFEB^{OE} HBs relative to CTRL. **d** HBs of the indicated genotypes undergo biliary differentiation upon transfer to matrigel coated plates as assessed by tubule formation after 24 hours. Scale bar 20 µm. **e** RT-PCR for cholangiocyte-specific markers 10 days after cholangiocyte differentiation (CTRL and TFEB^{OE} *n*=4 biological replicates; TFEB^{KO} *n*=3 biological replicates). Data are represented as mean ± SEM. $^{**}p \leq 0.01$; $^{***}p \leq 0.001$ two-way ANOVA. Source Data are provided as a Source Data file.

Supplementary Fig. 3

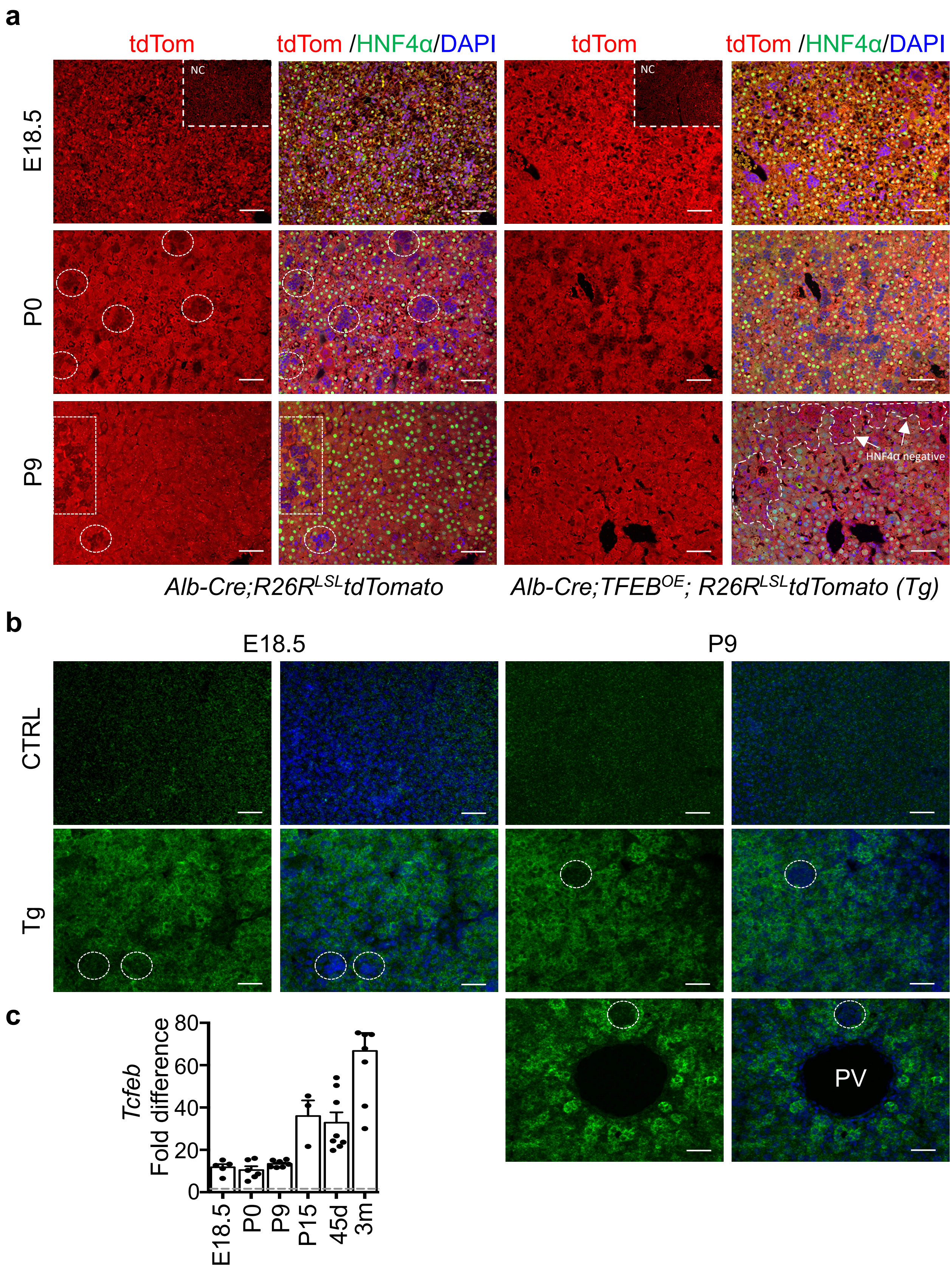

Supplementary Fig. 3. *Alb*-CRE induces TFEB overexpression in hepatocytes and precursor/cholangiocyte cells. **a** tdTom/HNFα dual immunofluorescence showing the efficiency of CRE recombinase at the indicated stages. NC=negative control. Dashed circles indicate tdTom negative cells. Scale bar 50 µm. **b** *In situ* hybridization analysis of *Tcfeb* in sections from E18.5 and P9 livers. Dashed circles indicate *Tcfeb* negative cells. Scale bar 50 µm. **c** Transcript levels of *Tcfeb* in livers isolated from TFEB liver-specific conditional overexpressing mice (Tg) at the indicated stages (E18.5,P0 *n*=6 biological replicates, P9 *n*=7 biological replicates, P15 *n*=3 biological replicates, 1m-3m *n*=8 biological replicates). *Tcfeb* mRNA levels were normalized to the age-matched control indicated by the dashed line. Data are represented as mean ± SEM. Source data are provided as a Source Data file.

Supplementary Fig. 4

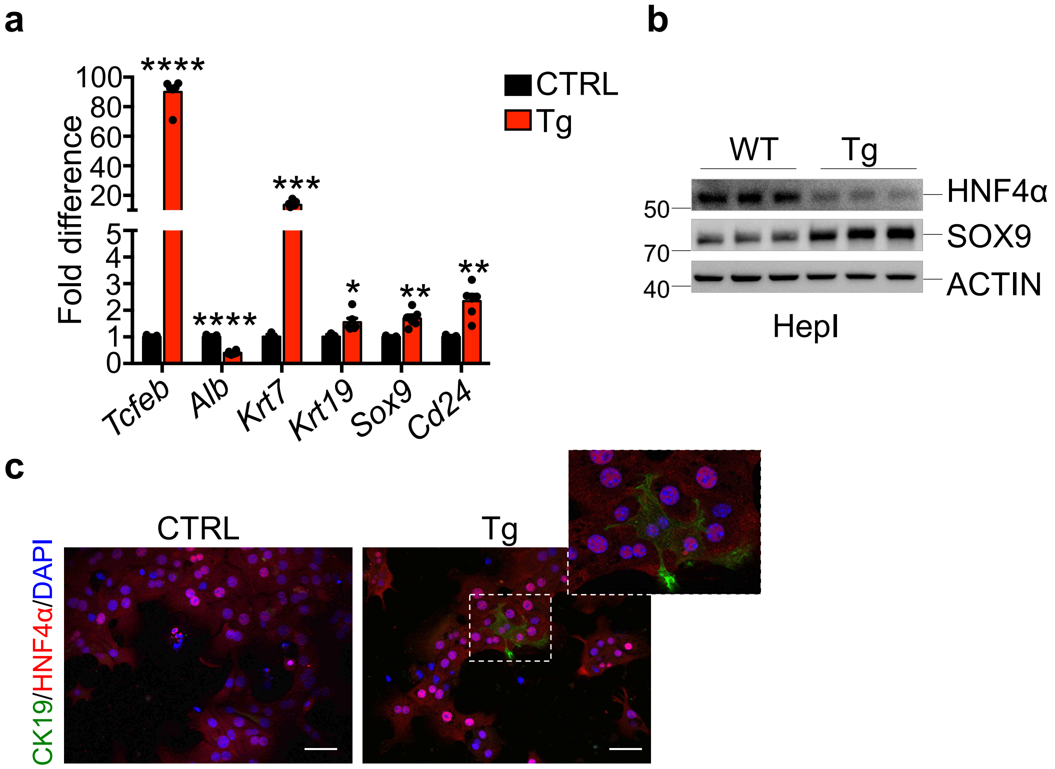

Supplementary Fig. 4. Hybrid features of TFEB^{OE} primary hepatocytes. **a** RT-PCR analysis of hepatocyte- and cholangiocyte- specific markers in primary hepatocytes isolated from Tg (*n*=6) and CTRL mice (*n*=3). Data are represented as mean ± SEM. **p* ≤ 0.05, ****p* ≤ 0.001, *****p* ≤ 0.0001 two-tailed Student's t-test. **b** Immunoblotting analysis of HNF4α and SOX9 in primary hepatocytes. GAPDH was used as loading control. **c** CK19/HNFα dual staining in primary hepatocytes of the indicated genotypes. Scale bar 20 µm. Source data are provided as a Source Data file.

Supplementary Fig. 5

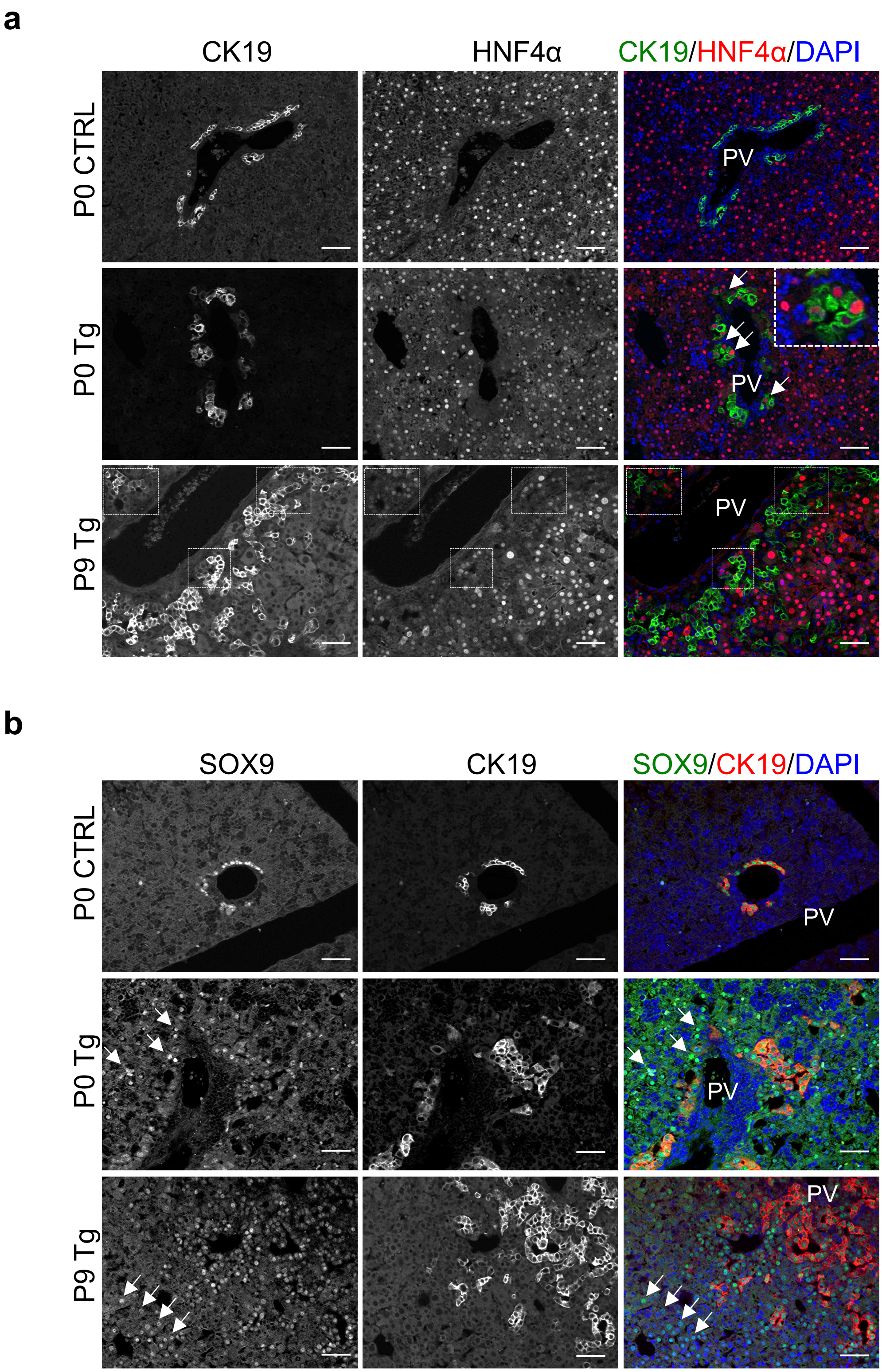

Supplementary Fig. 5. TFEB induces ductal/progenitor marker expression. **a,b** Immunofluorescence analysis for CK19/HNFα (**a**) and SOX9/CK19 (**b**) in P0 and P9 liver showing hybrid features of hepatic cells. Scale bar 50 μm.

Supplementary Fig. 6

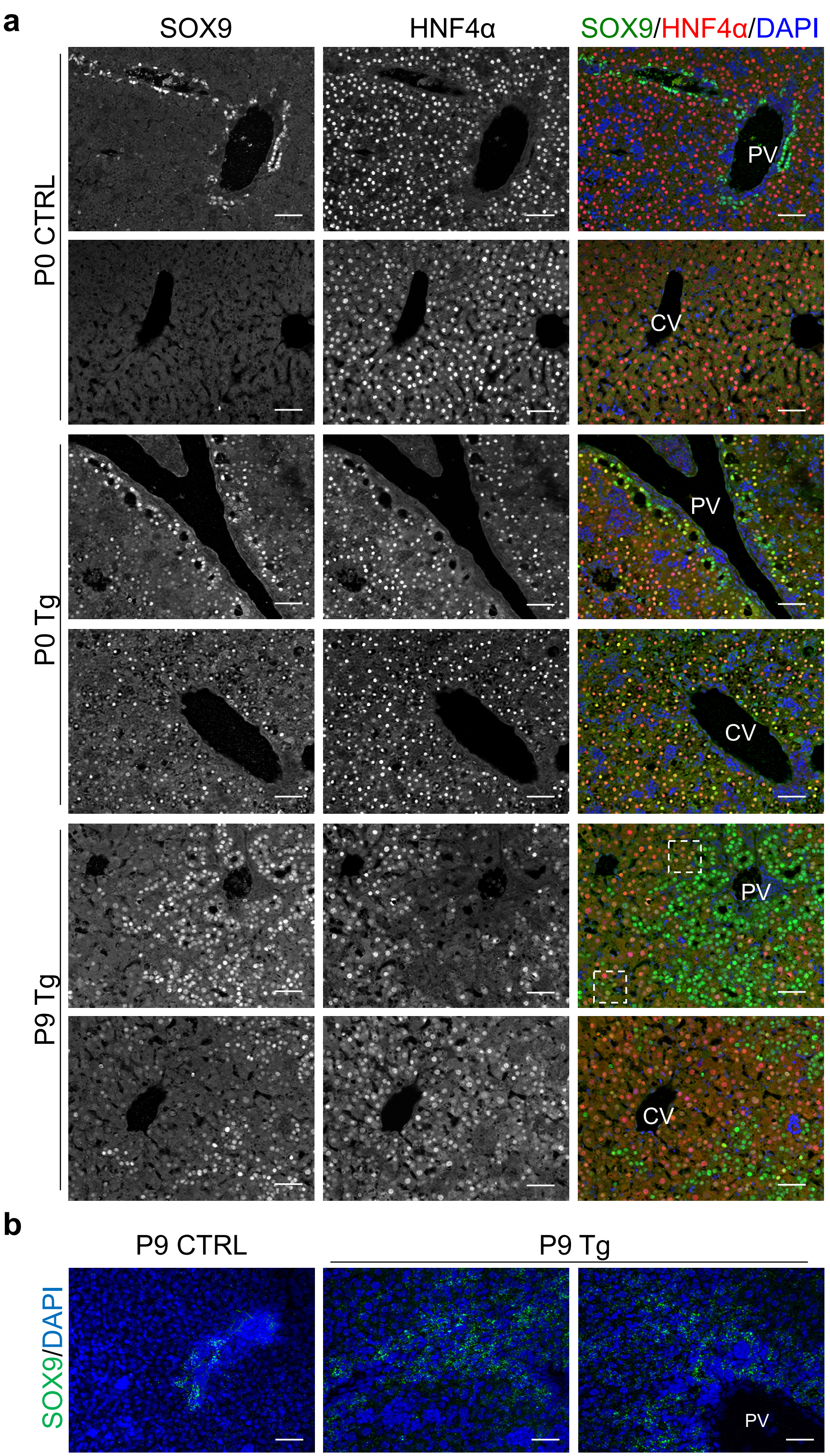

Supplementary Fig. 6. TFEB overexpression results in hepatocytes with hybrid features. **a** Immunofluorescence analysis for SOX9/HNFα in P0 and P9 liver Tg and CTRL showing the appearance of hybrid hepatocytes around both the central (CV) and the portal (PV) vein at P0 and expansion of $SOX9^+$ cells mainly around the portal area. Scale bar 50 μm. **b** *In situ* hybridization for SOX9 in CTRL and Tg P9 liver. Scale bar 50 μm.

Supplementary Fig. 7

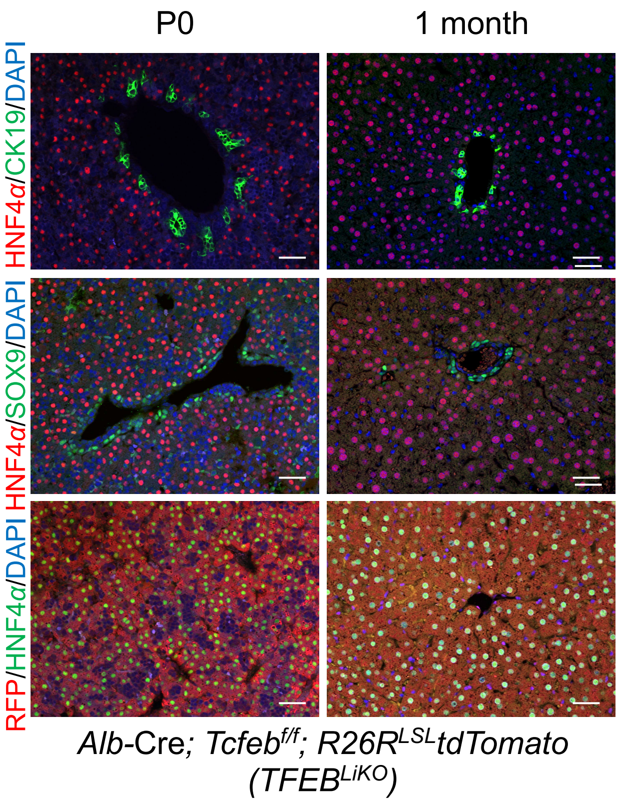

Supplementary Fig. 7. TFEB is dispensable for normal liver development. Immunofluorescence analysis of *Tcfeb*^{LiKO} liver at P0 and 1 month of age showing no cellular alteration upon TFEB depletion. Scale bar 100 µm.

Supplementary Fig. 8

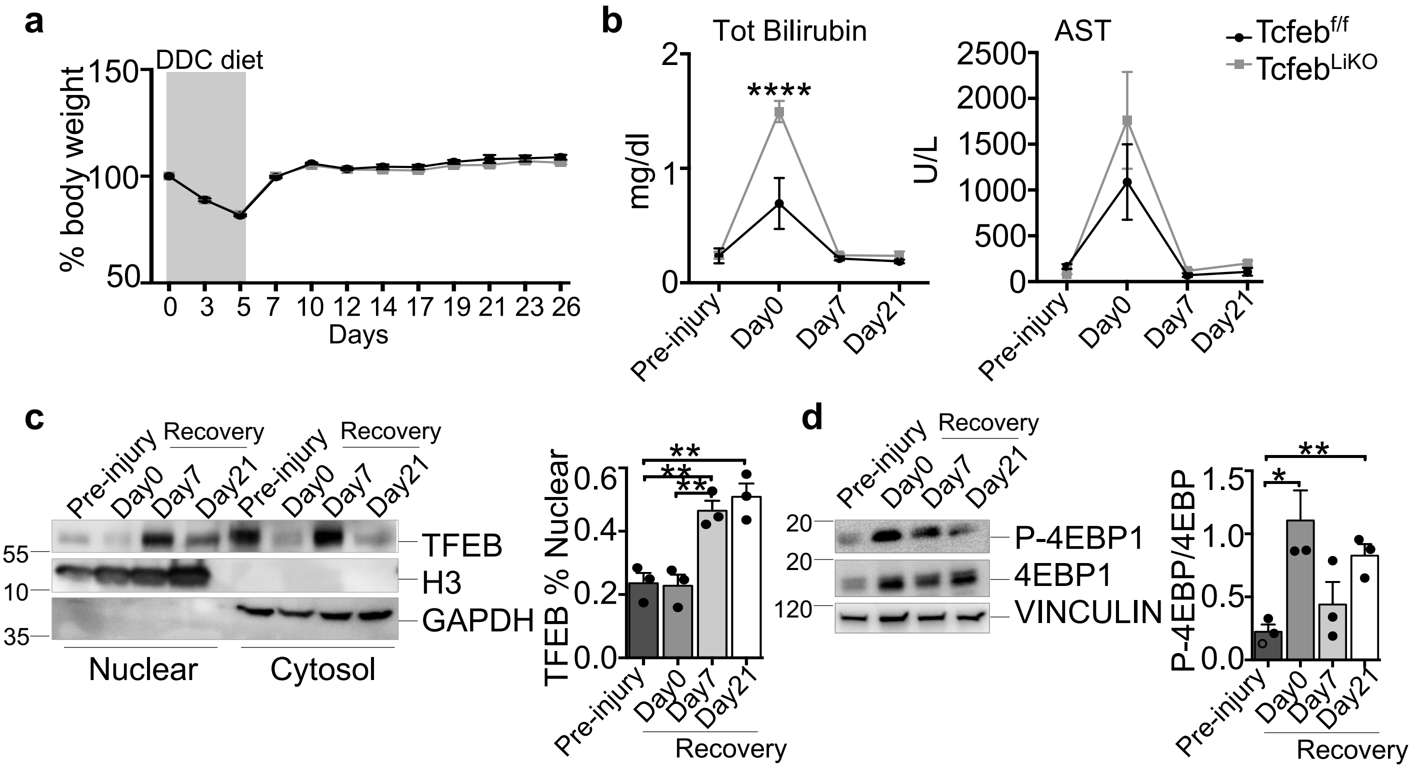

Supplementary Fig. 8. TFEB is activated during liver regeneration after injury. **a** Percentage of body weight during DDC diet and the subsequent recovery compared to T0 (*n*=6 biological replicates). Data are represented as mean ± SEM. **b** Serum analysis for markers associated with liver function (*n*=3 biological replicates). Data are represented as mean ± SEM. ****$p \leq 0.0001$ two-way ANOVA. **c** Nuclear/cytosolic fractionation in liver from mice treated with DDC-diet for 5 days and switched to a normal diet for 7 or 21 days with relative quantification (*n*=3 biological replicates). **d** Immunoblot analysis for 4EBP1 total and phosphorylated with relative quantification (*n*=3 biological replicates). Data are represented as mean ± SEM. *$p \leq 0.05$, **$p \leq 0.01$ two-tailed Student's t-test. Source data are provided as a Source Data file.

Supplementary Fig. 9

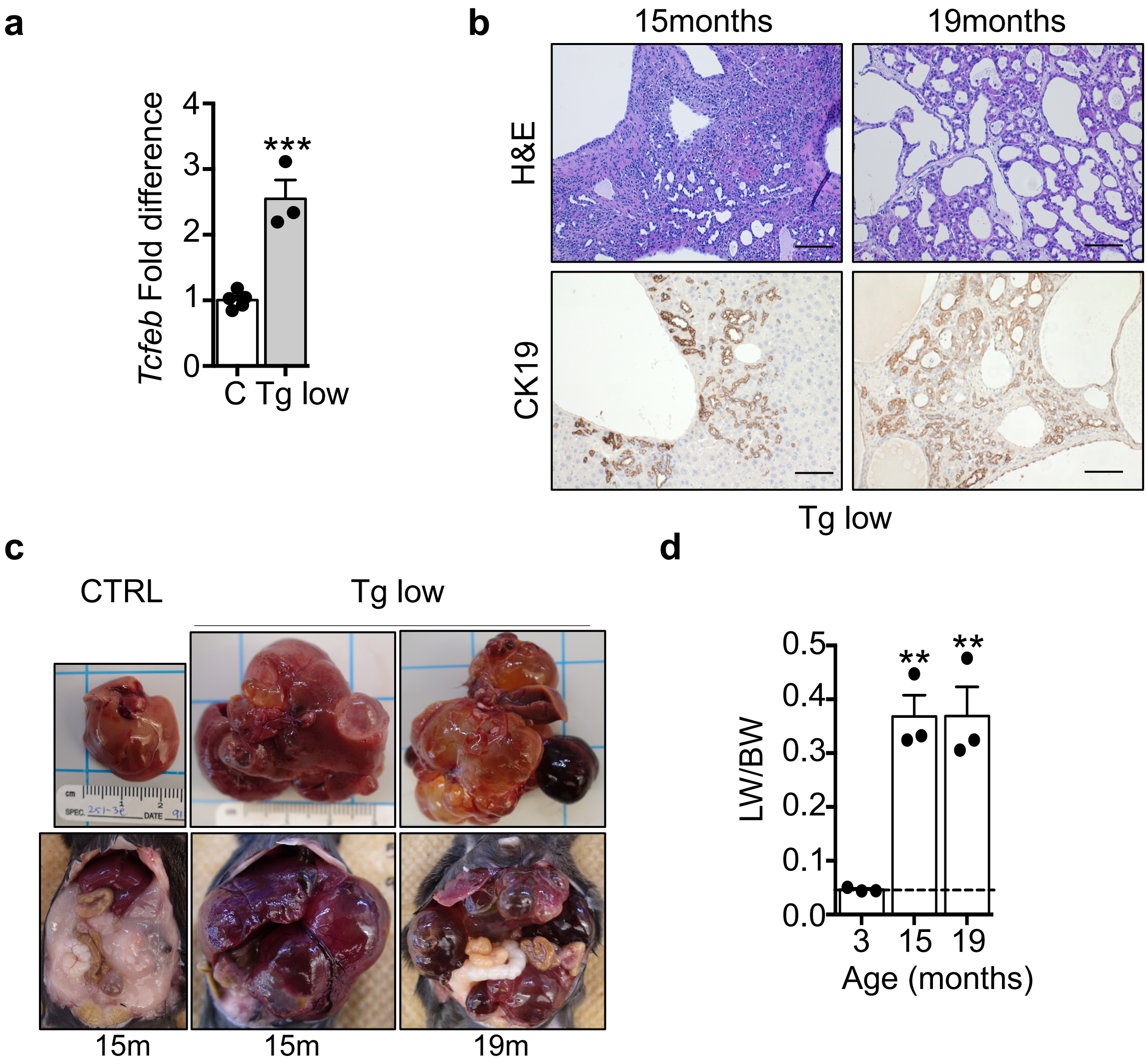

Supplementary Fig. 9. Characterization of 2.5-fold TFEB overexpressing mice. **a** Transcript levels of *Tcfeb* in livers isolated from control and TFEB liver-specific conditional overexpressing (2.5-fold compared to CTRL) mice (indicated as Tg) at lower levels at 3-months of age (CTRL *n*=5, Tg *n*=3 biological replicates). *Tcfeb* expression was normalized to liver from age-matched CTRL mice. **b** Histological characterization by H&E and CK19 immuno-staining of liver sections from Tg mice at 15 months. Scale bar 100 µm. **c** Gross appearance of liver from Tg low-expressing mice at indicated age. **d** Quantification of liver-to-body weight ratio in Tg mice at indicated age (*n*=3 biological replicates). Values were normalized to age-matched control liver (indicated by the dashed line). Data are represented as mean ± SEM. *$p \leq 0.05$, **$p \leq 0.01$, ***$p \leq 0.001$ two-tailed Student's t-test. Source data are provided as a Source Data file.

Supplementary Fig. 10

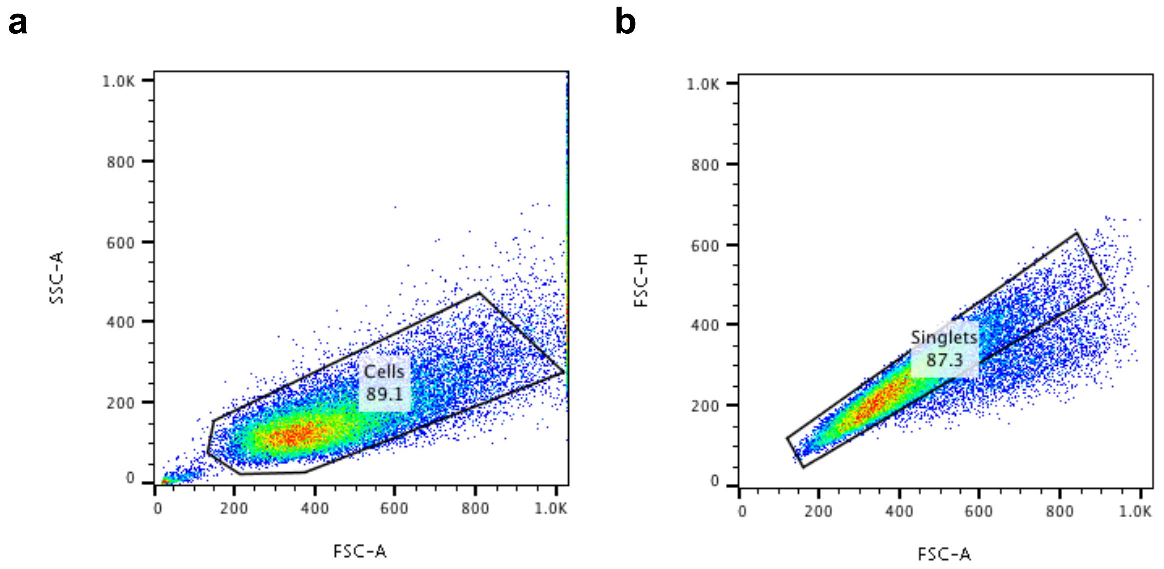

Supplementary Fig. 10. Gating strategy for FACS Analysis. **a** Cell debris and dead cells were excluded on a FSC-A/SSC-A plot. **b** Singlets were gated on a FSC-H/FSC-A plot.

Supplementary Fig. 11

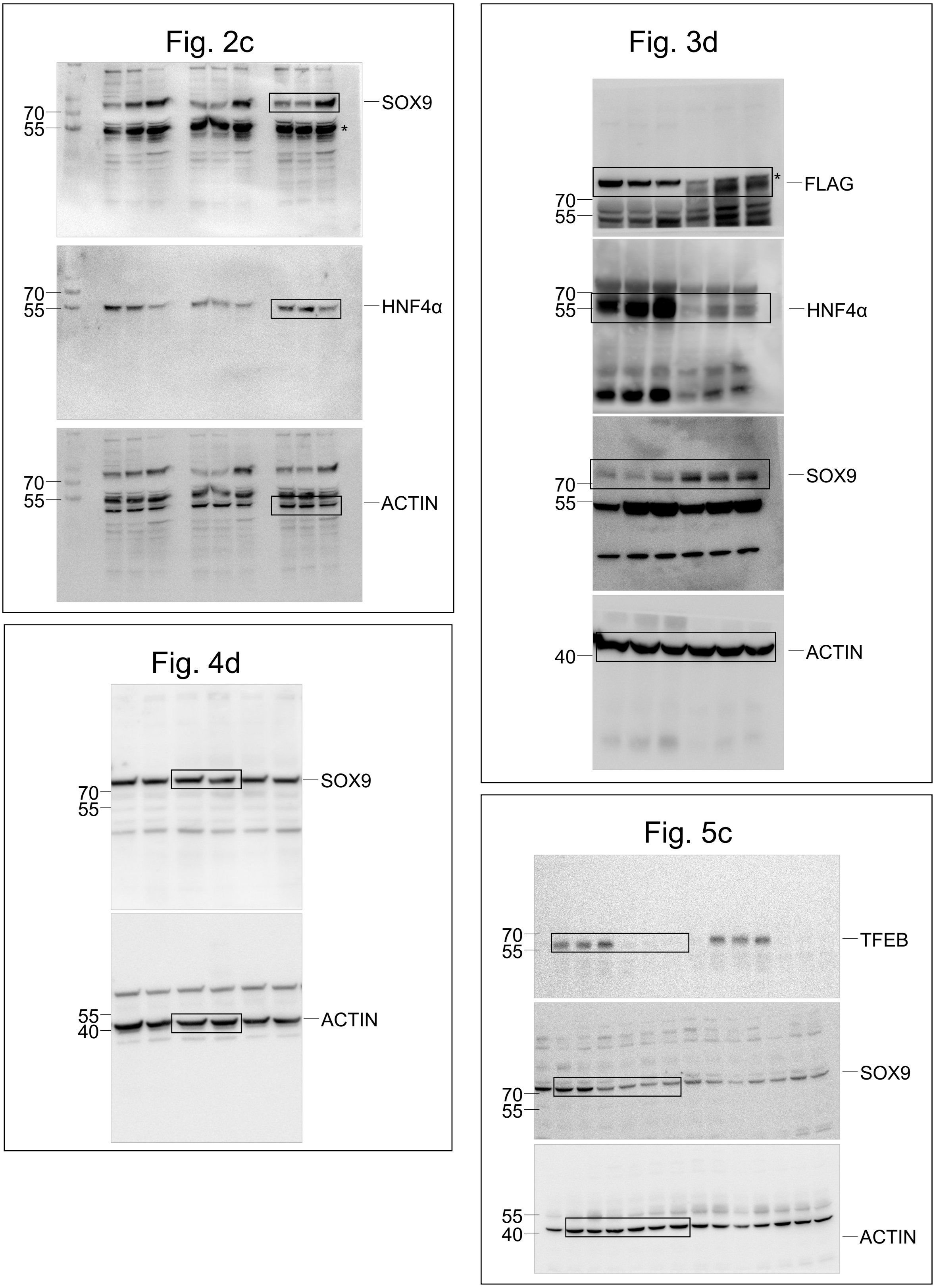

Supplementary Fig. 11. Uncropped original western blots represented in the main figures.

Supplementary Fig. 12

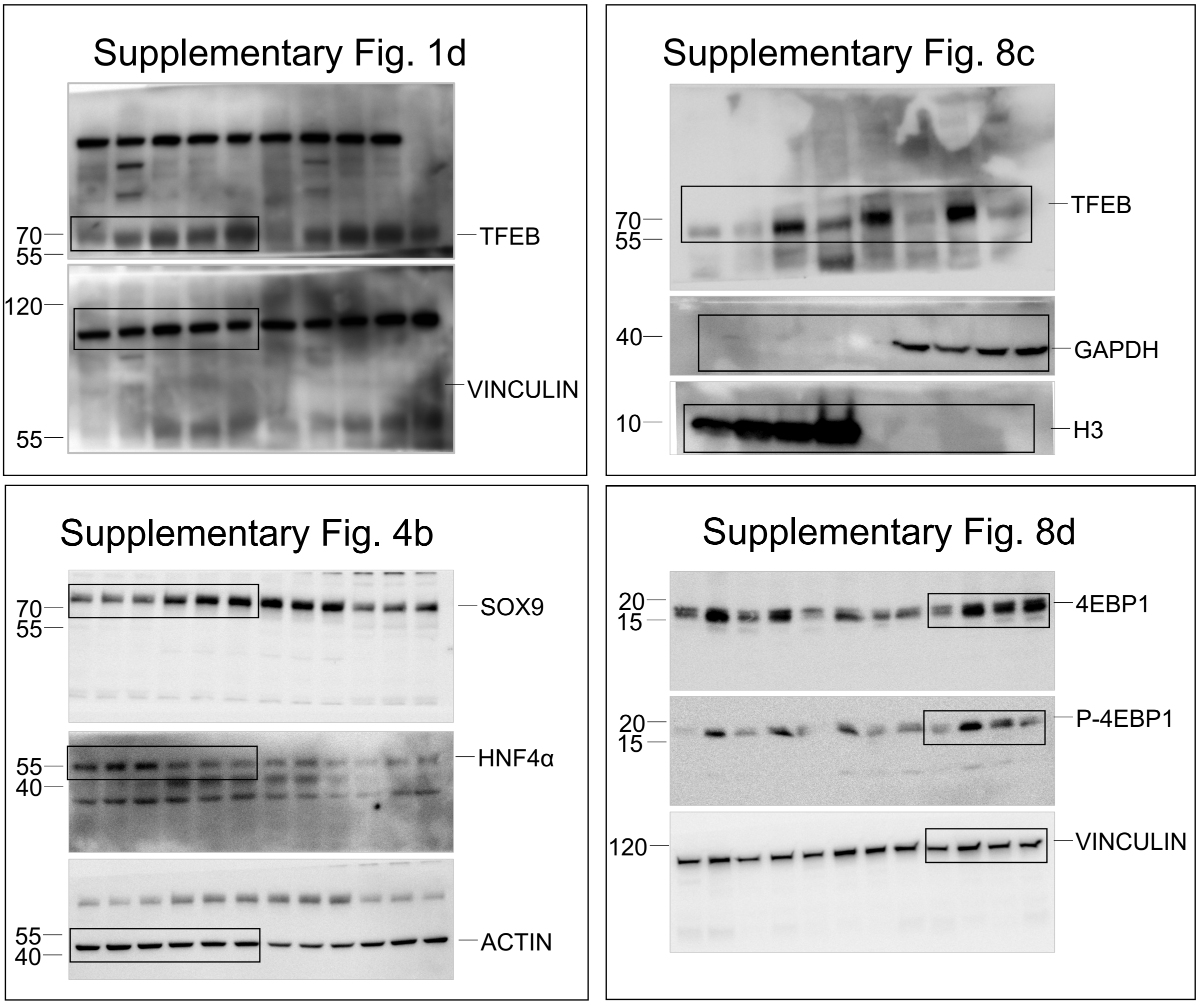

Supplementary Fig. 12. Uncropped original western blots represented in the supplementary figures.

Supplementary Tables

Supplementary Table 1. Gene-sets enriched in HB^{KO} versus HB ctrl 3 days after hepatocytic differentiation. Upregulated gene-sets identified by KEGG analysis (Fdr<0.05).

Geneset Details	*P* Value
Metabolism of xenobiotics by cytochrome P450	7.4883E-10
Chemical carcinogenesis	6.4016E-09
Drug metabolism - cytochrome P450	1.2116E-08
Retinol metabolism	1.5416E-08
Drug metabolism - other enzymes	6.298E-07
Fat digestion and absorption	6.0926E-06
Glutathione metabolism	1.2657E-05
Steroid hormone biosynthesis	0.00016116
PPAR signaling pathway	0.00021214
Pentose and glucuronate interconversions	0.00047401
Platinum drug resistance	0.0009436
Vitamin digestion and absorption	0.00156692
Ascorbate and aldarate metabolism	0.00235136
Arachidonic acid metabolism	0.00317744
Cholesterol metabolism	0.00351638

Supplementary Table 2. Gene-sets enriched in HB^{OE} versus HB ctrl 3 days after hepatocytic differentiation. Downregulated gene-sets identified by KEGG analysis (Fdr<0.05).

Geneset Details	*P* Value
Retinol metabolism	2.8403E-14
Calcium signaling pathway	0.00012288
Steroid hormone biosynthesis	1.5737E-11
Fatty acid metabolism	2.4197E-07
Bile secretion	2.022E-06
PPAR signaling pathway	4.1831E-05
Drug metabolism - other enzymes	0.00104415
Steroid biosynthesis	3.3995E-12
Fat digestion and absorption	3.8252E-07
Metabolism of xenobiotics by cytochrome P450	4.2611E-05
Fructose and mannose metabolism	6.7068E-05
Drug metabolism - cytochrome P450	0.00066233
Fatty acid degradation	0.00166059
Cholesterol metabolism	0.00604933

Supplementary Table 3. Gene-sets enriched in Tg versus CTRL P9 livers. Differentially expressed gene-sets identified by KEGG analysis (Fdr<0.05).

Down-regulated genes

Geneset Details	Count	*P* Value
Complement and coagulation cascades	53	2.055E-14
Peroxisome	44	6.8313E-10
Retinol metabolism	38	1.3504E-09
Steroid hormone biosynthesis	38	1.8191E-08
Chemical carcinogenesis	39	4.4993E-08
Tryptophan metabolism	25	6.7446E-07
Valine, leucine and isoleucine degradation	29	1.7841E-06
Linoleic acid metabolism	22	1.8624E-05
Cholesterol metabolism	25	4.2713E-05
Tyrosine metabolism	19	7.1767E-05
Glyoxylate and dicarboxylate metabolism	18	9.211E-05
Fatty acid degradation	22	9.6939E-05
Glycine, serine and threonine metabolism	20	9.8285E-05
Drug metabolism - cytochrome P450	23	0.00018933
Metabolism of xenobiotics by cytochrome P450	23	0.00029241
Staphylococcus aureus infection	27	0.00030916
Primary bile acid biosynthesis	11	0.00043284
ABC transporters	20	0.00043936
PPAR signaling pathway	32	0.00056848
Drug metabolism - other enzymes	29	0.00069238
Ascorbate and aldarate metabolism	10	0.00115783
Circadian rhythm	14	0.00145774
Arginine biosynthesis	10	0.00236616
Primary immunodeficiency	17	0.00243523
Propanoate metabolism	15	0.0028497
Graft-versus-host disease	17	0.00359702
Vitamin digestion and absorption	12	0.00426932

Up-regulated genes

Geneset Details	Count	*P* Value
Oxidative phosphorylation	67	9.2206E-17
Parkinson disease	65	5.0126E-14
Huntington disease	78	1.4655E-12
Thermogenesis	82	5.643E-10
Alzheimer disease	70	3.2042E-09
Proteasome	29	3.3163E-09
RNA transport	61	1.9051E-08
Non-alcoholic fatty liver disease (NAFLD)	58	5.1805E-07
Ribosome	44	1.3959E-06

Spliceosome	48	8.1E-06
Cell cycle	49	1.0843E-05
Tight junction	57	1.558E-05
Citrate cycle (TCA cycle)	18	3.7937E-05
Ubiquitin mediated proteolysis	48	5.7477E-05
mRNA surveillance pathway	32	8.8127E-05
Hippo signaling pathway	55	0.00017307
Ribosome biogenesis in eukaryotes	27	0.00042318
Pyruvate metabolism	18	0.00054524
Cardiac muscle contraction	29	0.00103658
Endocytosis	71	0.00164938
Adherens junction	28	0.00236656
Carbon metabolism	40	0.00303879

Supplementary Table 4. Gene expression of selected genes between CTRL and Tg livers. Microarray expression data for selected genes of interest. Data are reported as mean and standard deviation.

Gene symbol	WT (n=3) Mean	s.d	Tg (n=3) Mean	s.d	FDR	Signed ratio
Hepatocyte						
Otc	6093.15	526.04	3484.06	313.23	5.090361E-08	-1.748864
Hgd	5700.84	1002.03	4048.53	617.32	0.71016	-1.408124
Cyp7a1	419.23	206.80	259.73	35.17	0.61953	-1.614106
Cyp1a2	2165.76	267.56	501.56	34.94	1.75E-13	-4.31803
Cyp2a12	6085.46	602.39	2255.59	172.86	1.09E-11	-2.6794
Cyp2b13	584.91	327.13	73.53	8.29	1.08E-09	-7.95402
Cyp2b9	1552.05	301.50	249.47	10.40	6.00E-14	-6.22121
Cyp2c54	1194.49	566.34	115.57	37.63	2.70E-10	-10.3351
Pck1	13.33	1.6	8.59	0.23	6.96E-05	-1.550789
F9	1625.29	121.15	778.14	109.56	1.84E-08	-2.088672
Fah	6115.26	778.73	2959.96	84.88	1.19E-09	-2.065991
Tdo2	959.17	50.93	79.42	9.98	0	-12.07582
Hnf4a	133.94	20.24	96.38	5.8	0.0043	-1.389615
Agxt	13.326	1.60	8.593	0.235	6.9E-05	-1.550789
Ugt1a1	4813.46	418.84	1579.09	246.66	5.2E-11	-3.048237
Ugt2a3	576.66	226.91	109.102	45.210	3.48E-08	-5.26559
Adh1	13045.3	1444.30	8046.27	1128.07	4.28E-07	-1.697134
Arg1	5082.49	493.56	2958.95	385.59	5.43E-07	-1.71766
Apoc3	6747.28	249.99	2588.7	982.90	8.96E-08	-2.60643
Cebpa	901.61	92.52	517.16	46.148	6.66E-07	-1.74338
G6pc	5030.07	249.81	2815.94	163.02	6.55E-09	-1.78628
Hpd	7949.68	1310.82	3714.71	322.05	3.60E-09	-2.14005
H6pd	2191.29	99.75	1612.02	40.58	0.000166	-1.35934
Slc37a4	1690.99	96.16	928.29	88.90	2.92E-08	-1.82161
Progenitor/cholangiocyte						
Sox9	55.39	11.19	256.11	40.63	4.45E-11	4.623193
Sox4	206.05	19.681	728.06	72.84	5.68E-12	3.533408
Spp1 (Opn)	1711.06	251.75	2800.59	477.89	2.42E-05	1.636756
Krt7	69.974	19.69	152.065	26.58	1.71E-05	2.173152
Epcam	211.276	134.74	717.783	74.985	1.74E-06	3.397364
Itga3	91.199	7.73	120.123	12.26	0.010723	1.317157
Afp	20450.8	302.03	26100.2	546.5	2.21E-07	1.276237
Cd24	633.077	90.613	1043.43	108.95	6.87E-06	1.648187
Jag1	32.183	5.105	54.167	2.66	6.48E-05	1.683095
Yap1	126.084	1.148	163.497	14.98	0.005833	1.296725
Ctgf	121.200	18.993	268.33	65.30	3.14E-06	2.213956
Vim	370.95	66.546	637.811	126.33	6.65E-05	1.719405
Sprr1a	29.558	5.296	222.172	114.88	1.24E-09	7.516478
Tgfb2	38.28	4.69	78.39	15.99	8.24E-06	2.047589

Cldn4	63.287	21.71	1504.25	343.14	1.01E-13	23.76854
Cldn7	131.504	91.38	1351.01	196.32	5.98E-10	10.28129

Supplementary Table 5. Serum chemistry for CTRL and Tg mice. Serum analysis in P15 and 3-month-old CTRL and Tg mice and the relative *p* value calculated by Student two-tailed t- test.

	**P15**			**3months**		
	**WT**	**Tg**	***p value***	**WT**	**Tg**	***p value***
**ALT**	56.2±21.3	56.2±34.3	0.6422	25±1.7	86±15.5	0.00069
**AST**	163±25.3	259.8±111.4	0.095	83.7±29.5	220±8.5	0.00014
**ALP**	488.2±47.3	481.4±52.4	0.834	77±6.2	170.5±2.1	1.4E-05
**LDH**	1291.8±165.1	1814.25±403.6	0.032219984	162±29.7	473.5±190.2	0.02438
**BILIRUBIN**	0.078±0.01	0.25±0.14	0.02757	0.03±0.01	0.085±0.007	0.0081
**CHOLESTEROL**	107.4±21.4	162.6±31.5	0.0119	118.3±16.1	223.5±54.4	0.0431
**TOT BILE ACIDS**	0.192±0.123	1.24±0.24	0.0011	0.049±0.02	0.539±0.17	0.0008

Supplementary Table 6. Primers used for RT-PCR analysis.

Gene		Sequences (5'->3')
TFEB (Human/mouse)	forward	GGTGCAGTCCTACCTGGAGA
	reverse	GTGGGCAGCAAACTTGTTCC
Alb	forward	GCTGAGGCCATGTGCACCTCC
	reverse	CCATCAAGCTTCGGGGTCAGG
Afp	forward	AGCTTCCACGTTAGATTCCTCC
	reverse	ACAAACTGGGTAAAGGTGATGG
Hnf4α	forward	TGCCTGCCTCAAAGCCAT
	reverse	CACTCAGCCCCTTGGCAT
Arg1	forward	CATTGGCTTGCGAGACGTAGAC
	reverse	GCTGAAGGTCTCTTCCATCACC
Ttr	forward	CACCAAATCGTACTGGAAGACA
	reverse	GTCGTTGGCTGTGAAAACCAC
AldoB	forward	TGTCTGGAGGTATGAGTGAGG
	reverse	CTGGGTTGCCTTCTTGTTTGC
Otc	forward	GGATAGGGGATGGGAACAAT
	reverse	TCTGGCTCATAACCCTTTGG
Pepck	forward	GGCGATGACATTGCCTGGATGA
	reverse	TGTCTTCACTGAGGTGCCAGGA
Aat	forward	GGAGCAAACTCTCAGCAAGG
	reverse	ATGGACAGTCTGGGGAAGTG
Krt7	forward	CACCCGGAATGAGATTGCG
	reverse	GCACGCTGGTTCTTCAAGGT
Krt19	forward	TGCTGGATGAGCTGACTCTG
	reverse	AATCCACCTCCACACTGACC
Sox9	forward	CGAGGAAGATAAGTTCCCCGTGTGC
	reverse	CAGCAGCCTCCAGAGCTTGCCC
Sox4	forward	GACAGCGACAAGATTCCGTTC
	reverse	GTTGCCCGACTTCACCTTC
Cd24	forward	GTTGCACCGTTTCCCGGTAA
	reverse	CCCCTCTGGTGGTAGCGTTA
Sprr1	forward	TTGTGCCCCCAAAACCAAG
	reverse	GGCTCTGGTGCCTTAGGTTG
Ggt1	forward	CTTGTGCGAGGTGTTCTGC
	reverse	GCATAGTCACCGTCTCTCCTT
β2-microglobulin	forward	TGGTGCTTGTCTCACTGACC
	reverse	GTATGTTCGGCTTCCCATTC
S16	forward	AGGAGCGATTTGCTGGTGTGG
	reverse	GCTACCAGGGCCTTTGAGATG

Supplementary Table 7. Primary antibodies used for immuno-blots and stainings.

Antigen	Species in which the Ab was raised	Source	Cat#	Dilution
TFEB	Rabbit	Bethyl Laboratories	A303-673A	IF 1:200 WB 1:1000
FLAG	Mouse	Sigma	F1804	WB 1:1000
Ki67	Rabbit	Abcam	ab16667	IHC 1:200
CK19	Rabbit	Abcam	ab133496	IF/IHC 1:200
CK19-AlexaFluor^{647}	Rabbit	Abcam	ab192980	IF 1:200
BrdU	Rat	Abcam	ab6326	IF 1:200
HNF4α	Mouse	Abcam	ab41989	IF 1:200 WB 1:1000
VINCULIN	Mouse	Sigma	V9131	WB 1:1000
Beta-ACTIN	Mouse	Novus Biological	NB600-501	WB 1:1000
GAPDH	Mouse	Santa Cruz	sc-36062	WB 1:1000
SOX9	Rabbit	Millipore	AB5535	IF 1:200 WB 1:1000
RFP	Rabbit	Abcam	ab62341	IF 1:200 WB 1:1000
4EBP1	Rabbit	Cell signaling	9644	WB 1:1000
P-4EBP1 (Thr70)	Rabbit	Cell signaling	9455	WB 1:1000
H3	Rabbit	Cell signaling	4499	WB 1:3000

Supplementary Table 8: Primers used for ChIP-RT-PCR analysis.

Gene		Sequences (5'->3')
Sox9 site1	forward	AACAAACATTGCATGCCTCAG
	reverse	ACGTGAAGCAATCTCACAGGAT
Sox9 site2	forward	GTCAGGAGGCAAGAAGCAGAAC
	reverse	CGACCTTGAACTGACAAGTCCT
Sox9 site 3	forward	GAACTGCCTGGAAACTTCTGTG
	reverse	AGGGAAAACAGAGAACGAAACC
Sox9 site 4	forward	GGCAGCTGAGGGAAGAGGAG
	reverse	AAGGGGTCCAGGAGATTCATAC
Sox9 site 5	forward	GAGGAAGATAAGTTCCCCGTGT
	reverse	GTGGCTTGTTCTTGCTGGAG

Supplementary Table 9: Primers used for site-directed mutagenesis.

		Sequences (5'->3')
Sox9 ΔCLEAR1	forward	CAAGGTCGGCGTGGCCGACCGGCCGCAAG
	reverse	CTTGCGGCCGGTCGGCCACGCCGACCTTG
Sox9 ΔCLEAR2	forward	GAGAGACGAGGTGGGCCCCGGTTTCGTTC
	reverse	GAACGAAACCGGGGCCCACCTCGTCTCTC
Sox9 ΔCLEAR3	forward	GTCCGCGTCCGGGGAGGGAAGAGGAGCC
	reverse	GGCTCCTCTTCCCTCCCCGGACGCGGAC
Sox9 ΔCLEAR4	forward	GAACAAGCCACACGTGACCCATGAACGCCTTC
	reverse	GAAGGCGTTCATGGGTCACGTGTGGCTTGTTC

Supplementary Table 10: Primers used for AAV titer.

Name	Sequence (5'-3')	Target
AH-0003	TAAGTCCATGCTGGCAAGAGTG	TBG promoter
AH-0004	TCCTCTGCGAATGCATGTATAA	TBG promoter
EYFP_For2	GCATCGACTTCAAGGAGGAC	GFP
EGFP_Rev2	TGCACGCTGCCGTCCTCGATG	GFP
