# Supplementary material for: TFEB regulates murine liver cell fate during development and regeneration
Source: Nat Commun. 2020 May 18;11:2461. doi: 10.1038/s41467-020-16300-x (PMC7235048; doi:10.1038/s41467-020-16300-x)
Supplement: Supplementary file 2 — Reporting Summary [file 41467_2020_16300_MOESM2_ESM.pdf]

nature research

Corresponding author(s): Nunzia Pastore, Andrea Ballabio

Last updated by author(s): 03/04/2020

Reporting Summary

Nature Research wishes to improve the reproducibility of the work that we publish. This form provides structure for consistency and transparency in reporting. For further information on Nature Research policies, see Authors & Referees and the Editorial Policy Checklist .

Statistics

For all statistical analyses, confirm that the following items are present in the figure legend, table legend, main text, or Methods section.

n/a	Confirmed	
☐	☒	The exact sample size (n) for each experimental group/condition, given as a discrete number and unit of measurement
☐	☒	A statement on whether measurements were taken from distinct samples or whether the same sample was measured repeatedly
☐	☒	The statistical test(s) used AND whether they are one- or two-sided<br>*Only common tests should be described solely by name; describe more complex techniques in the Methods section.*
☒	☐	A description of all covariates tested
☒	☐	A description of any assumptions or corrections, such as tests of normality and adjustment for multiple comparisons
☐	☒	A full description of the statistical parameters including central tendency (e.g. means) or other basic estimates (e.g. regression coefficient) AND variation (e.g. standard deviation) or associated estimates of uncertainty (e.g. confidence intervals)
☒	☐	For null hypothesis testing, the test statistic (e.g. F, t, r) with confidence intervals, effect sizes, degrees of freedom and P value noted<br>*Give P values as exact values whenever suitable.*
☒	☐	For Bayesian analysis, information on the choice of priors and Markov chain Monte Carlo settings
☒	☐	For hierarchical and complex designs, identification of the appropriate level for tests and full reporting of outcomes
☒	☐	Estimates of effect sizes (e.g. Cohen's d, Pearson's r), indicating how they were calculated

Our web collection on statistics for biologists contains articles on many of the points above.

Software and code

Policy information about availability of computer code

Data collection	Zen black was used for image acquisition in Zeiss axiocam MR microscope. FACSDiva was used for the acquisition of FACS data.
Data analysis	CRISPRscan was used for the design of sgRNA sequences. Flow-Jo software was used for the analysis of FACS data. ImageJ software was used for image analysis. Bbduk software (bbmap suite) was used to trimm sequence reads. STAR 2.6.0a on mm10 reference assembly was used for sequence alignment. Htseq count 0.9.1 and Ensemble assembly were used to determine the expression levels of genes. EdgeR was used for differential expression analysis. Bioconductor R package Affy was used to process and normalize microarray data. Bioconductor R package clusterProfiler was used for enrichment analysis. MatchPWM algorithm with Biostrings package was used for the analysis of TFEB binding sites. GraphPad Prism v7 was used for statistical analysis.

For manuscripts utilizing custom algorithms or software that are central to the research but not yet described in published literature, software must be made available to editors/reviewers. We strongly encourage code deposition in a community repository (e.g. GitHub). See the Nature Research guidelines for submitting code & software for further information.

Data

Policy information about availability of data

All manuscripts must include a data availability statement. This statement should provide the following information, where applicable:

- Accession codes, unique identifiers, or web links for publicly available datasets
- A list of figures that have associated raw data
- A description of any restrictions on data availability

The data that support the findings of this study are available from the corresponding authors upon reasonable request. Sequencing data are available in NCBIs Gene Expression Omnibus (GEO) and are accessible through GEO Series accession number GSE35015.
The source data underlying Figs 2b,2c,2f, 3c, 4a-d, 5b-d, 5f, 6b, 7b, 8c, 8e, Supplementary Figs 1c, 1d, 2e, 3c, 4a, 8a-d, 9a, 9d are provided as a Souce Data file.

Field-specific reporting

Please select the one below that is the best fit for your research. If you are not sure, read the appropriate sections before making your selection.

- [x] Life sciences
- [ ] Behavioural & social sciences
- [ ] Ecological, evolutionary & environmental sciences

For a reference copy of the document with all sections, see nature.com/documents/nr-reporting-summary-flat.pdf

Life sciences study design

All studies must disclose on these points even when the disclosure is negative.

Sample size	No sample size calculation was performed. Appropriate samples sizes were used to determine statistical significance by Student's t-test or ANOVA. Sample size was chosen based on observed variability in preliminary assays and was determined to be adequate based on the consistency of the results between replicates.
Data exclusions	Data were not excluded from the analyses.
Replication	Replication numbers are indicated in the figure legends.
Randomization	No randomization was possible for animal experiments since mice were selected by genotype. All control animals were littermate controls. For experiments involving cells, all control and treatment wells were plated at the same time.
Blinding	Blinding was not possible for mouse experiments since experimental groups were determined based on the different genotypes. Automated methods of data recording and analysis were adopted when possible to prevent user bias.

Reporting for specific materials, systems and methods

We require information from authors about some types of materials, experimental systems and methods used in many studies. Here, indicate whether each material, system or method listed is relevant to your study. If you are not sure if a list item applies to your research, read the appropriate section before selecting a response.

Materials & experimental systems

n/a	Involved in the study	
[ ]	[x]	Antibodies
[ ]	[x]	Eukaryotic cell lines
[x]	[ ]	Palaeontology
[ ]	[x]	Animals and other organisms
[x]	[ ]	Human research participants
[x]	[ ]	Clinical data

Methods

n/a	Involved in the study	
[x]	[ ]	ChIP-seq
[ ]	[x]	Flow cytometry
[x]	[ ]	MRI-based neuroimaging

Antibodies

Antibodies used

Primaries:
Rb-anti-TFEB (Bethyl Laboratories) A303-673A 1:1000 for WB, 1:200 for IF
Mouse-anti-Flag (Sigma) F1804 1:1000 for WB
Rb-antiKi67 (Abcam) ab16667 1:200
Rb-antiCk19 (Abcam) ab133496 1:200 for IF
Rb-anti-CK19-Alexafluor647 (Abcam) ab192980 1:200 for IF
Rat-anti-BrdU (Abcam) ab6326 1:200 for IF
mouse-anti-HNF4a (Abcam) ab41989 1:1000 for WB, 1:200 for IF
mouse-anti-Vinculin (Sigma) V9131 1:1000 for WB
mouse-anti-B-actin (Novus Biological) NB600-501 1:1000 for WB
mouse-anti-Gapdh (Santa Cruz) sc-365062 1:1000 for WB
Rb-anti-Sox9 (Millipore) AB5535 1:1000 for WB, 1:200 for IF
Rb-anti-RFP (Abcam) ab62341 1:200 for IF
Secondary:
ThermoFisher AlexaFluor488 Goat anti-Rabbit A-11034 1:800
ThermoFisher AlexaFluor555 Goat anti-Mouse A32727 1:800
ThermoFisher AlexaFluor488 Goat anti-Rat A-11006 1:800
Anti-rabbit IgG HRP-linked (GE Healthcare Life science) NA934 1:3000
Anti-mouse IgG HRP-linked (GE Healthcare Life science) NXA931 1:3000

Validation

Rb-anti-TFEB (Bethyl Laboratories A303-673A), #Citations: 75 (CiteAb link): https://www.citeab.com/antibodies/658113-a303-673a-tfeb-antibody?utm_campaign=Widget+All+Citations&utm_medium=Widget&utm_source=Bethyl+Laboratories&utm_term=Bethyl+Laboratories. Validation from manufacturer related to application in our study: validated and approved for immunohistochemistry, immunoprecipitation and western blot. Validation from our study: gives band at expected molecular weight in WB analysis, gives no signal in TFEB KO samples in IF analysis.
Monoclonal Mouse-anti-Flag M2 (Sigma F1804), #Citations: 3357 (SigmaAldrich link): https://www.sigmaaldrich.com/catalog/product/sigma/f3165?lang=it&region=IT. Validation from manufacturer related to application in our study: validated and approved for immunoflorescence, ELISA, ChIP, electron mucroscopy, flow citometry, supershift assay, immunohistochemistry, immunoprecipitation and western blot. Validation from our study: gives band at expected molecular weight in WB analysis.
Rb-antiKi67 (Abcam ab16667), #Citations: 960 (Abcam link): https://www.abcam.com/ki67-antibody-sp6-ab16667-references.html#top-942. Validation from manufacturer related to application in our study: validated and approved for immunohistochemistry, immunofluorescence, flow citometry and western blot. Validation from our study: gives expected results in a control slide for IHC analysis.
Rb-antiCk19 (Abcam ab133496), #Citations: 24 (abcam link): https://www.abcam.com/cytokeratin-19-antibody-epncir127b-ab133496-references.html#top-916. Validation from manufacturer related to application in our study: validated and approved for immunohistochemistry and western blot. Validation from our study: gives expected results in a control slide for IHC analysis.
Rb-anti-CK19-Alexafluor647 (Abcam ab192980). Validation from manufacturer related to application in our study: validated and approved for immunofluorescence and flow citometry. Validation from our study: gives expected results in a control slide for IF analysis.
Rat-anti-BrdU (Abcam ab6326), #Citations: 989 (abcam link): https://www.abcam.com/brdu-antibody-bu175-icr1-proliferation-marker-ab6326-references.html#top-558. Validation from manufacturer related to application in our study: validated and approved for immunohistochemistry, immunofluorescence and flow citometry. Validation from our study: gives expected results in a control slide for IF analysis.
Mouse-anti-HNF4a (Abcam ab41989), #Citations: 55 (abcam link): https://www.abcam.com/hnf-4-alpha-antibody-k9218-chip-grade-ab41898-references.html#top-858. Validation from manufacturer related to application in our study: validated and approved for western blot, ELISA, immunoprecipitation, immunohistochemistry, ChIP and flow citometry. Validation from our study: gives expected results in a control slide for IF analysis and gives band at expected molecular weight in WB analysis.
Mouse-anti-Vinculin (Sigma V9131), #Citations: 721 (SigmaAldrich link): https://www.sigmaaldrich.com/catalog/search?term=V9131&interface=All&N=0&mode=match%20partialmax&lang=it&region=IT&focus=papers. Validation from manufacturer related to application in our study: validated and approved for western blot, immunohistochemistry and immunofluorescence. Validation from our study: gives band at expected molecular weight in WB analysis.
Mouse-anti-B-actin (Novus Biological NB600-501), #Citations: 388 (Novusbio link): https://www.novusbio.com/products/beta-actin-antibody-ac-15_nb600-501#PublicationSection. Validation from manufacturer related to application in our study: validated and approved for western blot, ELISA, flow citometry, immunohistochemistry, immunocitochemistry, ChIP and immunofluorescence. Validation from our study: gives band at expected molecular weight in WB analysis.
Mouse-anti-Gapdh (Santa Cruz sc-365062), #Citations: 818 SantaCruz link): https://www.scbt.com/p/gapdh-antibody-g-9. Validation from manufacturer related to application in our study: validated and approved for western blot, ELISA, immunohistochemistry and immunofluorescence. Validation from our study: gives band at expected molecular weight in WB analysis.
Rb-anti-Sox9 (Millipore AB5535), #Citations: 152 (Merk link): https://www.merckmillipore.com/IT/it/product/Anti-Sox9-Antibody,MM_NF-AB5535?ReferrerURL=https%3A%2F%2Fwww.google.com%2F#anchor_REF. Validation from manufacturer related to application in our study: validated and approved for western blot, ChIP, ChIP-seq, immunohistochemistry and immunofluorescence. Validation from our study: gives band at expected molecular weight in WB analysis and gives expected results in a control slide for IF analysis.
Rb-anti-RFP (Abcam ab62341), #Citations: 151 (Abcam link): https://www.abcam.com/rfp-antibody-ab62341-references.html#active-tab. Validation from manufacturer related to application in our study: validated and approved for western blot, immunohistochemistry, immunoprecipitation and immunofluorescence. Validation from our study: gives band at expected molecular weight in WB analysis and gives expected results in a control slide for IF analysis.

Eukaryotic cell lines

Policy information about cell lines

Cell line source(s)

Mouse Hepatoblasts were isolated from mouse embryos at stage E13.5
Primary mouse hepatocytes were isolated from 2-months old mice

Authentication

Cells were authenticated by analysis of specific cell markers.

Mycoplasma contamination

Primary cells used in the study were not tested for mycoplasma.

Commonly misidentified lines
(See ICLAC register)

No commonly used cell lines were used in the study

Animals and other organisms

Policy information about studies involving animals; ARRIVE guidelines recommended for reporting animal research

Laboratory animals	Male mice were mainly used in this study. Ages varied from Embryos to 6-months old mice as indicated in the text. TcfebLacZ/LacZ, Tcfebflox/flox and Tcfeb-3xFlagfs/fs transgenic mouse line generation has been previously described. Wild-type, Sox9flox/flox, R26RLSLtdTomato, Albumin-Cre and Krt19CreERT mouse lines were obtained from the Jackson laboratory (Bar, Harbor, ME).
Wild animals	No wild animals were used in the study.
Field-collected samples	No field-collected samples were used in the study.
Ethics oversight	All experiments were approved by the Baylor College of Medicine Institutional Animal Care and Use Committee (IACUC) and conform to the legal mandates and federal guidelines for the care and maintenance of laboratory animals.

Note that full information on the approval of the study protocol must also be provided in the manuscript.

Flow Cytometry

Plots

Confirm that:

- [x] The axis labels state the marker and fluorochrome used (e.g. CD4-FITC).
- [x] The axis scales are clearly visible. Include numbers along axes only for bottom left plot of group (a 'group' is an analysis of identical markers).
- [x] All plots are contour plots with outliers or pseudocolor plots.
- [x] A numerical value for number of cells or percentage (with statistics) is provided.

Methodology

Sample preparation	Cells were harvested with trypsin-EDTA and washed in PBS. For cell cycle analyses, cells were fixed in cold 70% ethanol and then washed in PBS. after treatment with RNAseA, cells were stained with Propidium Iodide and incubated at room temperature before the analysis.
Instrument	LSRII cytometer (BD Bioscience)
Software	FACSDiva (BD Bioscience) and Flow-Jo (Flow-Jo, LLC, Ashland, OR).
Cell population abundance	In all experiments at least 10000 events were acquired
Gating strategy	Cell debris and dead cells were excluded from FSC-A/SSC-A dot-plots as reported in supplementary Figure 11a.

- [x] Tick this box to confirm that a figure exemplifying the gating strategy is provided in the Supplementary Information.
